# Letter regarding article ‘Freezing of gait associated with a corpus callosum lesion’

**DOI:** 10.1186/s40734-016-0041-z

**Published:** 2016-09-10

**Authors:** Halil Onder

**Affiliations:** Department of Neurology, Hacettepe University Hospital, Sıhhıye, Ankara Post Code: 06010 Turkey

**Keywords:** Freezing of gait, Diffusion tensor imaging, Corpus callosum, Psychogenic movement disorders

## Abstract

Here, I discuss the report by Dale et al. and present some relevant comments, hoping that it will allow a better understanding of the patient’s situation as well as freezing of gait phenomenon. I will also discuss other disorders for differential diagnosis those should be kept in mind.

## To the Editor

I read the recent report by Dale et al. with great interest which described an atypical patient with freezing of gait (FOG) and associate FOG in the patient with the lesion of the anterior corpus callosum (CC) [1]. The patient had been suffering from a gradually progressive gait abnormality over four years and neurological examinations had revealed strictly asymmetrical FOG episodes (prominent in right foot). It has also been noted that upper extremity functioning was normal and other extra-pyramidal findings (tremor, rigidity, bradykinesia, postural imbalance) were absent including in the lower extremities. Based on the DTI results revealing the interhemispheric fiber loss in regions of CC and conventional MRI showing chronic lesion in CC, authors associated FOG episodes to CC lesion and rather postulated the patient as frontal gait disorder.

I appreciate the authors for presenting of this interesting patient, however I would like to comment the article in some aspects. First, as they stated, I agree that near complete resolution of FOG episodes by rolling walker was atypical for PD, but more interestingly and in contrast, no improvement had been achieved by other visual cues such as stepping over lines or the examiner’ s foot. Visual cues are often reported as being helpful in alleviating FOG [2] and have been suggested to benefit via correcting and regulating the scale as well as the amplitude generation problems associated with FOG [3]. Other explanations are their probable utility on focusing attention on stepping and [4] their compensation effect of proprioceptive and visual working memory deficits in patients with FOG [5]. On the other hand, although the severity of FOG may differ among distinct parkinsonian syndromes (which rather occur severely and earlier in PSP, MSA and pure akinesia according to PD); underlying mechanisms as well as the phenomenological occurrence of FOG cannot be explained divergently among differing diseases. In a crucial report, Lewis and Barker suggested that FOG develops due to the interruption of common neural networks along different points by a variety of pathological mechanisms [6]. Based on these knowledge, I think that totally contrasting responses to these two cues (Rolling walker-stepping over lines) in the reported patient by Dale et al. is also atypical and cannot be elucidated mechanistically by our current knowledge of FOG.

Another important point is that, the authors explain the mechanism of FOG by the hypothesis of disturbed connections of SMA, pre-SMA and dorsal premotor cortex. They refer the known function of SMA in the stage of controlling anticipatory postural adjustments [7]. I agree that there is an increasing evidence for the association of FOG with SMA which are discussed based on by its role in anticipatory postural adjustment [7, 8]. Nonetheless, as postural balance and the function of anticipatory postural adjustment are closely related with each other in the mechanistic way; asserting SMA connectivity disturbances as the responsible pathway would also be irrational in this patient whose postural sway and standing balance were totally normal. Hence, I think that evaluating gait disorder in this patient as an organic pathology may even be challengeable. Psychogenic gait disorders are common presentations of psychogenic movement disorders; and interestingly, balance of these patients has also been suggested to be rather preserved as in this case in recent years [9]. Of note; in accordance with this thought, the patient had not been suffering falls, despite severe FOG episodes. Gathering these knowledge, I think that the gait disorder in this patient may be evaluated under the subgroup of psychogenic movement disorders. Additively, in my opinion, the disease of primary progressive freezing of gait (PPFG) may be another possible diagnosis in this case. Although normal postural sway and lack of falls decrease this possibility, long lasting PPFG patients have been reported in literature [10]. I think clinical follow-up of the patient will probably give crucial data regarding this argument.

In conclusion, I think this report still constitutes a considerably rare and interesting presentation, however reevaluation of the patient based on these comments and certainly psychiatry consultation will provide a better understanding of the underlying pathomechanisms. Additively, I would like to emphasize that, MRI had also revealed several periventricular lesions beside lesion of CC which might also cause- additional connectivity disruptions of distinct brain regions. Hence, I think that associating FOG with solely abnormalities of CC and its connections based on a single case as in this report may be questionable and may cause misleading views.

## Authors’ response

Marian L. Dale, Martina Mancini and Fay B. Horak

We describe a case of freezing of gait and atypical “floor scanning” in a patient without Parkinson’s disease. Objective measures showed significant asymmetry of right and left lower leg angular velocity during forward walking, such that for every step with her left foot, she took one to three small steps with her right foot. Gait improved dramatically with certain cuing mechanisms (rolling walker, trekking poles) but not others (stepping over lines or the examiner’s foot), but that is not implausible. Both in practice, and as reported in the literature, some patients with freezing of gait respond differentially to various sensory cues- visual, auditory, or proprioceptive [11, 12].

The differential response to cuing mechanisms with intact postural sway in no way suggests a psychogenic movement disorder. In fact, this patient’s movements were stereotyped and not distractable. Specifically, her performance actually worsened with dual task during walking (gait speed was 0.58 m/s for single task and 0.54 m/s with dual task; turning duration was 4 s for the single task and 5.2 s for the dual task). In addition, a newly diagnosed psychogenic movement disorder would be less likely in the elderly. The fact that the patient did not fall in the home is also not evidence of elaboration of symptoms or a psychogenic movement disorder. At home she constantly used her rolling walker as a compensatory mechanism, to the point that she was even able to perform chores such as cleaning the ceiling with a Swiffer while using the walker. Certainly fear of falling could have contributed to a superimposed cautious gait pattern, but this is not unlike many patients with freezing of gait and organic disease.

On two points we agree with Dr. Onder: 1) primary progressive freezing of gait remains on the differential pending the evolution of her neurological exam, and 2) the particular combination of periventricular, centrum semiovale and anterior callosal lesions likely resulted in the abnormal gait pattern. Indeed, the fact that the centrum semiovale lesions were modestly worse on the left may explain the asymmetry of the floor scanning pattern. Nonetheless, the lesion in the anterior corpus callosum was the most apparent, and the importance of lesions in the genu of the corpus callosum for frontal gait disorders is increasingly recognized (Wang, 2012; Fling, 2016) [13, 14].

## Notes

See related case report by Dale et al. 10.1186/s40734-016-0030-2

## Abbreviations

DTI, diffusion tensor imaging; MRI, magnetic resonange imaging; PD, Parkinson’s disease; pre-SMA, presupplementary motor area; SMA, supplementary motor area
